# Persistence of frequently transmitted drug-resistant HIV-1 variants can be explained by high viral replication capacity

**DOI:** 10.1186/s12977-014-0105-9

**Published:** 2014-11-29

**Authors:** Marieke Pingen, Annemarie MJ Wensing, Katrien Fransen, Annelies De Bel, Dorien de Jong, Andy IM Hoepelman, Emmanouil Magiorkinis, Dimitrios Paraskevis, Maja M Lunar, Mario Poljak, Monique Nijhuis, Charles AB Boucher, On behalf of the SPREAD programme

**Affiliations:** Department of Virology, Viroscience Lab, Erasmus MC, Postbus 2040, 3000 CA Rotterdam, the Netherlands; Virology, Department of Medical Microbiology, University Medical Center Utrecht, Utrecht, the Netherlands; Institute of Tropical Medicine, Antwerpen, Belgium; AIDS Reference Laboratory of the Vrije Universiteit Brussel, subunit Universitair Ziekenhuis Brussels, Brussels, Belgium; Internal Medicine and Infectious Diseases, University Medical Center Utrecht, Utrecht, the Netherlands; National Retrovirus Reference Center, Department of Hygiene, Epidemiology and Medical Statistics, Athens, Greece; Institute of Microbiology and Immunology, Faculty of Medicine, University of Ljubljana, Ljubljana, Slovenia

**Keywords:** HIV, Drug resistance, Transmission, Evolution, Reversion, Persistence, Compensatory fixation

## Abstract

**Background:**

In approximately 10% of newly diagnosed individuals in Europe, HIV-1 variants harboring transmitted drug resistance mutations (TDRM) are detected. For some TDRM it has been shown that they revert to wild type while other mutations persist in the absence of therapy. To understand the mechanisms explaining persistence we investigated the *in vivo* evolution of frequently transmitted HIV-1 variants and their impact on *in vitro* replicative capacity.

**Results:**

We selected 31 individuals infected with HIV-1 harboring frequently observed TDRM such as M41L or K103N in reverse transcriptase (RT) or M46L in protease. In all these samples, polymorphisms at non-TDRM positions were present at baseline (median protease: 5, RT: 6). Extensive analysis of viral evolution of protease and RT demonstrated that the majority of TDRM (51/55) persisted for at least a year and even up to eight years in the plasma. During follow-up only limited selection of additional polymorphisms was observed (median: 1).

To investigate the impact of frequently observed TDRM on the replication capacity, mutant viruses were constructed with the most frequently encountered TDRM as site-directed mutants in the genetic background of the lab strain HXB2. In addition, viruses containing patient-derived protease or RT harboring similar TDRM were made. The replicative capacity of all viral variants was determined by infecting peripheral blood mononuclear cells and subsequently monitoring virus replication. The majority of site-directed mutations (M46I/M46L in protease and M41L, M41L + T215Y and K103N in RT) decreased viral replicative capacity; only protease mutation L90M did not hamper viral replication. Interestingly, most patient-derived viruses had a higher *in vitro* replicative capacity than the corresponding site-directed mutant viruses.

**Conclusions:**

We demonstrate limited *in vivo* evolution of protease and RT harbouring frequently observed TDRM in the plasma. This is in line with the high *in vitro* replication capacity of patient-derived viruses harbouring TDRM compared to site-directed mutant viruses harbouring TDRM. As site-directed mutant viruses have a lower replication capacity than the patient-derived viruses with similar mutational patterns, we propose that (baseline) polymorphisms function as compensatory mutations improving viral replication capacity.

## Background

The viral enzymes reverse transcriptase (RT) and protease were the first targets of antiretroviral therapy and the most commonly used drug regimens still aim at inhibiting these viral proteins [1]. In resource-rich settings, drug resistance mutations in protease and RT are detected in 10-15% of newly diagnosed HIV patients [2,3].

The majority of transmitted drug-resistant viruses contain limited resistance profiles to single drug classes. Nucleoside RT inhibitor (NRTI) mutations are the most frequently observed transmitted drug resistance mutations (TDRM). Especially thymidine analogue mutations (TAMs) M41L and T215 variants, that have been selected by drugs extensively used in the past, are often observed in newly diagnosed patients [4]. A worrying trend is the increased prevalence of non-nucleoside RT inhibitor (NNRTI) related mutations in newly diagnosed patients [3,5], as single NNRTI mutations, such as the frequently observed K103N mutation, can result in high levels of resistance against first generation NNRTIs [6]. In protease, M46I/L and L90M are the most frequently observed TDRM [2,3]. When present in combination with other protease drug resistance mutations, both M46I/L and L90M are related to reduced susceptibility to several protease inhibitors (PIs) [6].

It is generally acknowledged that most drug resistance mutations decrease the replicative capacity (RC) of HIV-1 [7,8]. As such, in the absence of drugs TDRM can revert to wild type, thereby increasing viral RC. Indeed, follow-up of untreated individuals diagnosed with a drug resistant HIV variant revealed that certain mutations with a detrimental effect on the viral RC, such as M184V in RT, after transmission to a new host often revert rapidly in the plasma [9,10]. In addition, the use of very sensitive assays shows that minority drug resistance mutations are frequently found in untreated individuals, suggestive of reversion after transmission [11,12].

However, follow-up of patients diagnosed with HIV-1 harboring TDRM has revealed that TAMs, PI- and NNRTI-related TDRM often persist for prolonged periods [10,13-25]. The mechanisms explaining persistence have not been fully resolved. Based on the available literature [13,15-25], we have previously proposed two possible mechanisms to explain persistence of TDRM [9]. When the effect of the TDRM on the RC is very small, reversion may take a very long time. Alternatively, when the TDRM decreases the RC considerably the presence or selection of additional compensatory mutations can prevent reversion of the TDRM.

The aim of our study was to gain more insight in the mechanisms causing persistence of drug resistant HIV-1 variants after transmission. Therefore, we investigated the molecular evolution of HIV-1 protease and RT harboring the most frequently observed TDRM in great detail. The majority of TDRM persisted during the follow-up, and only few additional polymorphisms were selected during this period. Most patient-derived viruses had a higher RC than the corresponding site-directed mutant viruses, indicating that persistence can be explained by a high replication capacity of most transmitted drug resistant HIV-1 variants.

## Results

### Patients diagnosed with a transmitted drug resistant HIV-1 variant

To investigate the *in vivo* evolution of transmitted drug resistant HIV variants, we selected 31 patients from four European countries (Belgium, Greece, the Netherlands, Slovenia) who were diagnosed in 2001 to 2008 with an HIV variant harboring a frequently observed TDRM (prevalence >5% in patients diagnosed with HIV-1 harboring TDRM in the SPREAD-programme). Patients were included if a plasma sample was available at one year (10–14 months) after diagnosis if therapy was not yet initiated. If available, a third time point before start of treatment was investigated. Prior negative HIV tests were available for 14 patients, revealing that at least nine patients had been infected for less than two years. The majority of the patients were men having sex with men (MSM), which is the most important route of transmission in Western Europe. The median plasma HIV-RNA in our group of patients was 4.6 log copies/ml, comparable to the median HIV-RNA observed in the SPREAD-programme in 2002–2006 (4.8 log copies/ml). The median baseline CD4 count was 653 cells/mm^3^, which is higher than the median observed in the SPREAD programme (343 cells/mm^3^) [3].

Surveillance studies demonstrated that most transmitted drug resistant HIV-1 variants harbor resistance against a single drug class [3,4]. In line with this observation, only 3/31 of the patients selected for this study had been diagnosed with an HIV-1 variant resistant to multiple drug classes. A total of 55 mutations at positions included in the WHO list for surveillance of transmitted drug resistant HIV-1 [26] were observed in the transmitted viruses at baseline. A single TDRM was detected in 10/16 patients with viruses harboring only NRTI-related TDRM, for the other six patients a profile of two to four TDRM was observed. The vast majority of NRTI-related TDRM were TAM-related mutations. In six of the selected patients viral variants containing a single NNRTI-related TDRM were observed. Six patients were diagnosed with HIV-1 harboring a single PI-related TDRM (Table 1). In addition to TDRM, polymorphisms were present in all baseline sequences. For variants containing RT TDRM, the median number of RT polymorphisms was 7 (range: 4–21) when compared to HXB2 and 6 to consensus B (range: 2–19). Viruses harboring PR resistance mutations had a median of 6 baseline polymorphisms in protease when compared to HXB2 (range: 4–9) and median of 5 when compared to consensus B (range: 3–8).

**Table 1:** **Patient characteristics, resistance mutations and evolution**

**ID**	**Gender**	**Last negative HIV test**	**Country of origin**	**Diagnosis**	**Risk group**	**Months after first analysis**	**Plasma HIV RNA** **(copies/ml)**	**CD4 count**	**Sub-type**	**Resistance Profile PR**	**Resistance Profile RT**	**p-distance**	**p-value dN/dS**
Transmitted variants harboring only NRTI-related mutations													
P01	Male		NL	May 2007	MSM	0	>750000	461	B		M41L		
						10					M41L	0.001	1.000
						16					M41L	0.002	0.290
P02	Male		NL	Jan 2008	MSM	0	21800	423	B		M41L		
						12					M41L	0.005	0.225
						28					M41L	0.005	0.225
P03	Male	Jan 2004	BE	Jun 2005	MSM	0	41000	483	B		M41L		
						11					M41L	0.000	1.000
						32					M41L	0.002	0.152
P04	Male		NL	Feb 2007	MSM	0	102000	322	B		L210LS		
						11					-	0.000	1.000
						25					-	0.002	1.000
P05	Male		SL	Jun 2001	MSM	0	12267	950	B		T215D		
						12					T215D	0.011	0.060
						99					T215D	0.007	0.428
P06	Male	Mar 2005	SL	Feb 2006	MSM	0	797000	953	B		T215S		
						14					T215S	0.000	1.000
						21					T215S	0.000	1.000
P07	Male		NL	Sep 2008	MSM	0	36300	521	B		T215D		
						11					T215D	0.000	1.000
						27					T215D	0.000	1.000
P08	Male	Sep 2004	NL	Dec 2004	MSM	0	583000	596	B		T215IT		
						13					-	0.000	1.000
P09	Male	Sep 2006	NL	Sep 2007	MSM	0	158000	678	B		T215AT		
						13					T215AT	0.001	0.294
						20					T215A	0.000	1.000
P10	Male	Oct 2003	NL	Jan 2005	MSM	0	89800	289	B		K219N		
						11					K219N	0.000	1.000
						44					K219N	0.000	1.000
P11	Male		BE	Mar 2006	HSX	0	318000	966	B		D67N T215C		
						13					D67N T215C	0.001	0.291
P12	Male		NL	Feb 2007	MSM	0	55900	609	B		D67G T215C K219E		
						11					D67G T215C K219E	0.007	0.156
						24					D67G T215C K219E	0.000	1.000
P13	Male	Jul 2004	NL	Nov 2007	MSM	0	294000	531	B		D67G T215C K219E		
						12					D67G T215C K219E	0.000	1.000
						14					D67G T215C K219E	0.000	1.000
P14	Male	Apr 2005	NL	Jun 2005	HSX	0	750000	577	B		D67G T215C K219E		
						14					D67G T215C K219E	0.000	1.000
P15	Male		NL	Aug 2005	MSM	0	81000	470	B		M41L T69S T210E T215ST		1.000
						11					M41L T69S T210DE T215ST	0.000	1.000
						39					M41L T69S T210E T215ST	0.000	1.000
						77					M41L T69S T210E T215ST	0.000	1.000
P16	Male	Mar 2005	NL	Jun 2006	MSM	0	34600	1129	B		M41L T69S T210E T215ST		
						13					M41L T69AS T210E T215ST	0.000	1.000
						33					M41L T69S T210E T215ST	0.001	1.000
						49					M41L T69S T210E T215ST	0.001	1.000
Transmitted variants harboring only NNRTI-related mutations													
P17	Male	Feb 2005	NL	Sep 2006	MSM	0	5990	790	B		K103N		
						12					K103N	0.000	1.000
						30					K103N	0.000	1.000
P18	Male	Jun 2004	BE	Apr 2006	MSM	0	39900	648	B		K103N		
						12					K103N	0.000	1.000
						28					K103N	0.000	1.000
P19	Male		NL	Sep 2005	MSM	0	21400	359	B		K103Q		
						12					K103Q	0.000	1.000
						59					K103Q	0.001	0.304
P20	Male	1995	SL	Sep 2005	MSM	0	29300	421	B		Y181C		
						11					Y181C	0.000	1.000
						49					Y181C	0.002	0.305
P21	Female		GR	Sep 2004	HSX	0	905	699	B		G190A		
						10			B		G190A	0.005	0.866
P22	Male		GR	Jun 2004	?	0	10500	918	B		G190A		
						13			B		G190A	0.005	0.387
Transmitted variants harboring only PI-related mutations													
P23	Male		NL	Apr 2007	HSX	0	700000	664	B	M46L			
						14				M46L		0.000	1.000
						22				M46L		0.000	1.000
P24	Male	Jan 2006	NL	Apr 2008	MSM	0	5170	742	B	M46L			
						10				M46L		0.001	0.310
						29				M46L		0.004	0.471
P25	Male	Jul 2005	NL	Aug 2008	MSM	0	421000	409	B	M46L			
						14				M46L		0.000	1.000
						23				M46L		0.000	1.000
P26	male		NL	Aug 2008	MSM	0	111000	657	B	M46L			
						14				M46L		0.000	1.000
						26				M46L		0.001	0.299
P27	male	05-11-04	NL	Apr 2007	MSM	0	18100	699	B	M46L			
						13				M46L		0.000	1.000
						38				M46L		0.001	0.306
P28	male	15-02-03	NL	Mar 2005	MSM	0	69000	480	B	L90M			
						13				L90M		0.000	1.000
Transmitted variants harboring mutations against two drug classes													
P29	male		NL	Dec 2001	MSM	0	288	1468	B		D67G Y181CY T215C K219E		
						10					D67G T215C K219E	0.006	0.148
						46					D67G T215C K219E	0.000	1.000
P30	male		NL	Jan 2005	HSX	0	26600	667	B	G73S L90M	K103N		
						12				L90M	K103N	0.001	0.306
						18				L90M	K103N	0.001	0.306
P31	female		GR	Jul 2004	HSX	0	696	1288	B	I54V V82A L90M	M41L D67N L210W T215D		
						10				F53FL I54V V82A L90M	M41L D67N L210W T215D	0.001	0.293

*Abbreviations*: *PR* protease, *RT* reverse transcriptase, *NRTI* nucleoside reverse transcriptase inhibitor, *NNRTI* non- nucleoside reverse transcriptase inhibitor, *PI* protease inhibitor, *BE* Belgium, *GR* Greece, *NL* the Netherlands, *SL* Slovenia, *HSX* heterosexual, *MSM* Men having sex with men? unknown route of transmission.

### *In vivo* evolution of transmitted drug resistant HIV-1 variants

The vast majority (51/55) of TDRM persisted during the first year of follow-up. For 24/31 patients a third and sometimes a fourth genotypic analysis was performed at a median of 28 months (range: 14–99 months) after the first sample. During this more extensive follow-up period of up to eight years, all resistance mutations present at one year after diagnosis persisted in the plasma (Table 1).

To gain more understanding of *in vivo* persistence of TDRM, we performed a comprehensive analysis of *in vivo* viral evolution during the follow-up. Viruses harboring protease drug-resistance mutations selected a median of 1 (range: 0–1) additional polymorphisms in protease during the first year of follow-up. Likewise, viruses harboring drug-resistance mutations in RT selected a median of 1 (range 0–3) additional RT polymorphisms (Table 2). As a measure of evolution at the nucleotide level, the p-distance between baseline and follow-up sequences was calculated. For the majority of patients, this revealed a very low p-distance between baseline and one year, confirming limited viral evolution. In line with this observation, the dN/dS ratio of the viral populations, which is an indicator of selection, did not change significantly in any patient (Table 2). However, in all transmitted viruses at least one change at a polymorphic site was observed, which is described in Table 2.

**Table 2:** **Evolution of transmitted drug resistant HIV variants**

**ID**	**Months after first sample**	**Protease amino acid 4-99**			**Reverse transcriptase amino acid 41-230**		
		**Baseline**	**Reversion**	**Additional mutations**	**Baseline**	**Reversion**	**Additional mutations**
Transmitted variants harboring only NRTI-related mutations							
P01	0	*S37N* L63P I93L			**M41L** V60I I135T S162C K166R R211G *L214F*		
	10					-K166R	+V106IV
	16						V106IV > I
P02	0	T12A K14KR Q18HQ L19IL *S37N* L63P I93L			**M41L** V60I I135T S162G K166EK I167F R211G *L214F*		
	12		-K14KR, −Q18HQ	L19IL > IKLQ		-S162G -I167F	K166EK > KR
	28		T12A > AT	L19IKLQ > IL		R211G > GR	+T165IT
P03	0	T12A I13V L19I *S37NS* I64V C67CR			**M41L** V60I F61FS *E122K* D123E I178L V179IV E203EG Q207EQ *L214F*		
	11		-S37NS -I64V > IV -C67CR	+I62IV		-F61FS -E203EG	+S162X V179IV > I Q207EQ > KQ + R211KR
	32		T12A > AT	I62IV > V		-S162X -I178L -V179I	Q207KQ > EQ
P04	0	E35D *S37D* D60E I62V L63P A71T I72V I93L			K49R V60I V118I *E122K* D123DE I135R S162D **L210LS** R211G		
	11			+I72V > EV		**-L210LS**	D123DE > E S162D > S162X + T200IT + E204EK
	25			+T12AT + K14KR + V77IV		-S162X, R211G > GR -E204EK	+T165IT
P05	0	*S37N* I64V			K64R R83K I178L I202V *L214F* **T215D**		
	12			+M36I		-K64R	+S68N + E122K
	99			+I13IV + K14KR + K45KR		R83K > KR, −I202V	+A158AS + S162T
P06	0	L10I K14EV *S37N* L63T E65EV I72T V77I I93L			E122K I142V D177E Q207E *L214F* **T215S**		
	14			K14EV > E E65EV > V		-E122K > EK	
	21						
P07	0	I15L L19V *S37N* R41K D60E L63P I72IV I93L			V60I S68G R83K V90I A98S *E122P* D123DEG I135L S162C D177E I202IV R211K *L214F* **T215D**		
	11			+M36IM		−202IV	D123DEG > DE
	27		-M36IM	L19V > IV			D123DE > DEG + T200IT
P08	0	L10I *S37N* R41K I62V L63S V77I I93L			V60I S68G *E122K* I135V S162NS T165IT Q174HQ G196E R211G *L214F* **T215IT**		
	13					-S162NS -T165IT**-T215IT**	Q174HQ > H
P09	0	*S37N* I62V L63T I64L V77I			S68T *E122K* I135V T139A G196E Q197R *L214F* **T215AT**		
	13					-T139A	+H198HR
	20			+R57KR		-H198HR	S68T > AT + T139AT + **T215AT > A**
P10	0	I15V E35D *S37D* D60E L63P V77I I93L			S68K T69N A98S L100LV *E122K* D123E I135R N136NT Q145E S162C I178M E194D I195L G196E T200A I202V Q207K R211G *L214F* **K219N** H221Y K223Q		
	11			+R41K		-L100LV -N136NT	
	44			+K45KR + R57KR		-I135R	+K49KR
P11	0	S37H R41KR R57K Q61D			V60I **D67N** T69E V106I D121Y I135T S162C D177E G196E E203D Q207E R211KR *L214F* **T215C**		
	13					-V106I L214F > FL	+T200IT
P12	0	L10I T12S L19I L63T			V60I **D67G** S68G I135T I178M R211KR *L214F* **T215C K219E**		
	11		-L10I	L19I > T		-R211KR	+E122EK
	24			+L10I L19T > I + I62IV		-E122EK -I135T	+Q207LQR + R211KR
P13	0	T12S L19I L63T I64IM			V60I **D67G** S68G A158S I178M *L214F* **T215C K219E**		
	12			I64IM > M			+E40Q
	14					-E40Q	
P14	0	T12S L19T L63T			V60I **D67G** S68G I135IT I178M *L214F* **T215C K219E**		
	14						+E122EK I135IT > T
P15	0	L19I E35D *S37N* R57KR L63P V77IV I93L			**M41LT69S** D86DE *E122K* S162C I178L E204DE Q207EKQ **L210E** R211K *L214F* **T215ST**		
	11		-R57KR	V77IV > I			Q207EKQ > KQR **L210E > DE**
	39		V77I > IV	*S37N > DN* + R57KR			+V60VI + I195IL Q207KQR > x L210DE > E R211K > KN
	77		’-R57KR			S162C > CS	E204DE > DEKNR211KN > K
P16	0	L19I E35D *S37NS* L63P V77IV I93L			**M41L T69S** D86E K104KR *E122K* S162C I178L E204DE Q207KQR **L210E** R211DEKN *L214F* **T215S**		
	13			I72IM		-K104KR	**T69S > AS** S162C > CW
	33		-I72IM	E35D > DEKN *S37NS > N* V77IV > I		-E204DE	**T69AS > S** S162CW > W + E194DE Q207KQR > R R211DEKN > D
	49			E35DEKN > DE		-E194DE	Q207R > QR R211D > DEKN
P17	1	E35D R41K L63P I93L			**K103N** *E122K* D123E R211K *L214F*		
	12						+K173KT + D177DN
	30			+*S37N*		-K173KT	+Q174HQ + Q207QR R211K > KQ
Transmitted variants harboring only NNRTI-related mutations							
P18	0	L10IV I13IV I15IV L19IL I62V L63PS I64LV C67S V77I			K64R **K103N** *E122K* D123E K173EK Q174QR V179I T200A R211K *L214F*		
	12		-L19IL	L10IV > I L63PS > X I64LV > V		-K173EK -Q174QR	+D177DN
	28						+R72RS + Q174QR
P19	0	L10I I15V *S37T* R41K C67G G68E H69R			V60I **K103Q** *E122K* D123E I142V R211K *L214F*		
	10						
	12						
	59			G68E > D			+ T200IT
P20	0	T12N K14R *S37N* R41KR I64V			*E122K* D123E I135T **Y181C** T200A I202V R211K *L214F*		
	11		S37N > NS				
	49		K14R > KR -R41KR	+E35D S37NS > N + L63HQ		E122K > EK I135T > IT	D123E > AE
P21	0	I13V *S37NT* L63P A71AG			I50N G51W P52A V60IV R83K A98AG K101H S105LS D177E V179I **G190A** R211K *L214F*		
	10		-A71AG	S37NT > NST		-I50N -G51Q -P52A -A98AG -S105LS	V60IV > I + E122K K173EK
P22	0	I13V M36T *S37N* L63P			S48Q R83KR K101H D123DE D177E V179I **G190A** *L214F* H235R		
	13			M36T > IMT		-S48Q -H235R	D123DE > DEKN + S162CS
P23	0	*S37N* **M46L** D60E I62V L63S I72V V77I I93L			K49R V60I V118I I135R E169D R211G *L214F*		
	14						+F87FL + E204EK
	22					V60I > IV -F87FL -E204EK	
P24	0	E35DE *S37N* **M46L** D60E I62V L63S I93L			K49KR V60I V118I *E122K* I135R R211G		
	10		-E35DE -I93L	+K70KR		-K49KR	
	29		-K70KR				+K104KR + S162C
P25	0	E35D *S37N* **M46L** D60E I62V L63S I93L			K49R V60I V118I *E122K* I135R R211G		
	14			+R41KR			+D123E
	23			L63S > PS I93L > IL			D123E > DEKN + I178ILV
Transmitted variants harboring only PI-related mutations							
P26	0	E35D *S37N* **M46L** D60E I62V L63S I93L			K49R V60I V118I *E122K* D123DN I135R R211G		
	14			+L19IL		-D123DN	+T165IT
	26			L19IL > X + A71AV			T165IT > I + E204EK
P27	0	E35D *S37N* **M46L** D60E I62V L63S I93L			K49R V60I V118I *E122K* I135R N136NT S162NS I167IT R211G		
	13			L63S > APS		-N136NT -S162NS -I167IT	
	38		-E35D	L63APS > A			
P28	0	L19T *S37N* L63P **L90M** I93L			*E122K*T200A *L214F* K220X		
	13		*S37N > NS*			-K220X	
Transmitted variants harboring mutations against two drug classes							
P29	0	T4IT T12S L19IV L63X			V60I **D67G** S68G K70KR I178M **Y181CY** *L214F* **T215C K219E**		
	10		-T4IT	+L10I L19IV > I L63X > T		-K70KR **-Y181CY**	+I135IT + E204EG + R211KR
	46		-L10I	T12S > PS + G16AG L19I > IV + M36IM L63T > PT + I64IV		-E204EG -R211KR	I135IT > T
P30	0	L10I I13V I15V I62V L63P **G73S L90M**			V60I A98S **K103N** D121Y D123E I135T R172KR *L214F*		
	12		-G73S			I135T > IT -R172KR	
	18			*+S37NS*			+K102KR
P31	0	L10I I15V K20R E35D M36I *S37N* **I54V** Q58E L63P A71V **V82A L90M**			**M41L** K43N V60I **D67N** *E122P* I135T E138A I142V **L210W** R211M *L214F* **T215D**		
	10			**+F53FL**		-L214F	+T139I + I178IV

Patient-derived sequences are compared to HXB2. **Bold** positions indicate positions related to drug resistance, *italics* indicate polymorphisms of HXB2 compared to consensus B.

### Impact of frequently observed TDRMs on *in vitro* RC

**Figure 1:**
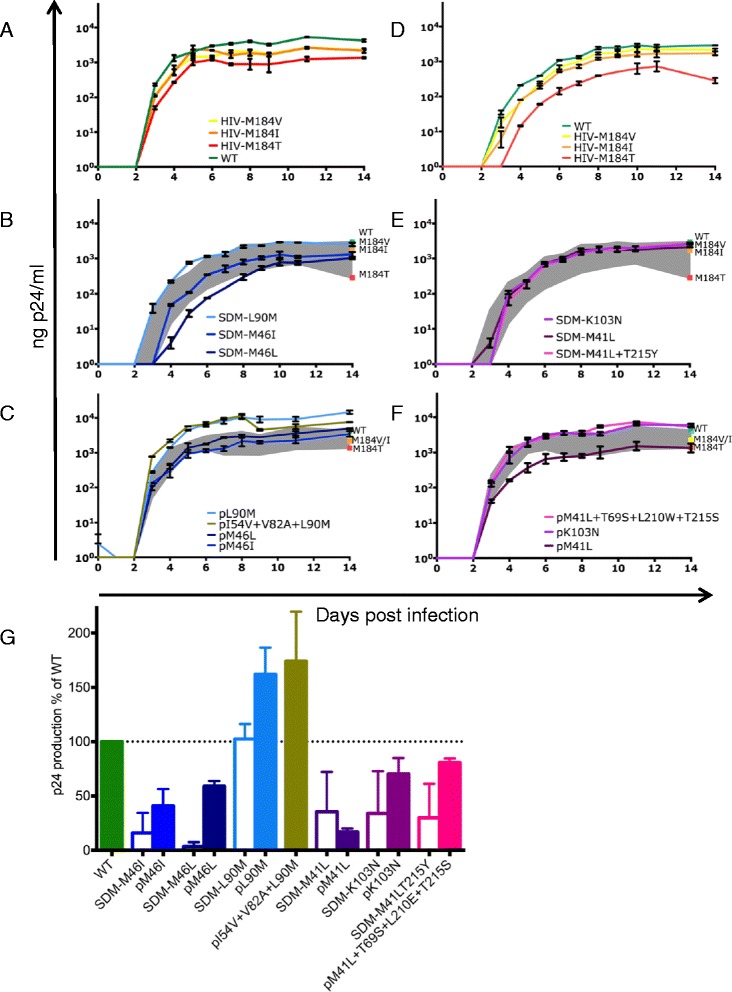
**Impact of frequently observed transmitted drug-resistance mutations on viral replicative capacity.** The replicative capacity of site-directed mutant (SDM) viruses and patient-derived viruses was determined by infecting donor peripheral blood mononuclear cells with equal amounts of viral p24. In all experiments, control viruses HIV-M184V, −M184I and –M184T and wild type (WT) HIV were used as reference viruses. Representative experiments are shown in **A**-**C** and **D**-**F**. Error bars indicate standard deviation (SD) of mean within one experiment. Four biological replicates were performed for all viruses. **(A-C)** Replicative capacity of SDM-viruses **(B, C)** compared to control viruses **(A)**. **(D-F)** Replicative capacity of patient-derived viruses **(E, F)** compared to control viruses **(D)**. RC of WT and control viruses **(A, D)** is indicated in the corresponding graphs by a square, and the range in RC of WT and M184T by the grey area. **(G)** The median p24 production of both experiments as a percentage of WT in the corresponding experiment for all protease or reverse transcriptase mutant viruses. Error bars indicate range (n = 4).

We determined the impact of TDRM on viral RC by introducing frequently observed drug-resistance mutations M46I, M46L or L90M in protease or M41L, M41L + T215Y or K103N in RT in the background of the lab strain HXB2 by site-directed mutagenesis (Figure 1). Viruses were named according to mutations and origin; the prefix “SDM” indicates site-directed mutagenesis. The RC of all viral variants was determined in primary peripheral blood mononuclear cells (PBMCs), which are natural target cells for HIV. Site-directed mutants HIV-M184V, −I and –T with a known impact on RC were used as controls, and to enable comparison of RC between various experiments [27]. The difference in RC between HIV-WT, −M184V and -M184I has been demonstrated to be biologically relevant *in vivo* [28,29].

All mutations caused a decrease in RC as compared to HIV-WT, except for mutation L90M in protease. The reduction in RC of the M41L, M41L + T215Y and K103N variants was comparable to each other, and to controls HIV-M184V and -I. M46I and M46L in protease resulted in the most severe reduction of RC (Figure 1).

### *In vitro* RC of patient-derived HIV-1 variants harboring frequently observed TDRM

Subsequently, the RC of frequently observed TDRM was determined in their natural genetic background (Figure 1). We constructed recombinant viruses using patient-derived protease containing M46L, M46I or L90M, or patient-derived n-terminus of RT containing M41L or K103N into HXB2. In addition, two more complex transmitted viruses were studied: a protease-variant containing I54V + V82A + L90M and an RT-variant carrying M41L + T69S + L210E + T215S. Patient-derived clones are indicated by the prefix “p”, followed by the TDRM.

The RC of p46I and p46L was similar to controls HIV-M184I and –V, indicating a diminished replication. The RT variant pK103N had an RC comparable to HIV-WT and the RC of pL90M was higher than HIV-WT. For M41L, it has been described that V60I and S162A function as compensatory mutations in transmitted HIV-1 variants [30]. We selected a patient-virus with M41L but without the potential compensatory mutations (pM41L). In this genetic background, the viral RC was as low as HIV-M184T and even lower than SDM-M41L. However, *in vivo* the variant containing this M41L mutation persisted for 8 months without selection of V60I or S162A before the patient initiated therapy (data not shown).

Interestingly, except for the pM41L variant, all patient-derived viruses had a higher RC than the corresponding site-directed mutants (Figure 1). The RC of all protease mutation-harboring patient-derived viruses was higher than the corresponding SDM-viruses, and the RC of pL90M and pI54V + V82A + L90M were even higher than WT. In line with these results, the RC of pK103N and pM41L + T69S + L210E + T215S surpassed the RC of the corresponding SDM-viruses to the level of wild type virus. These observations suggest the presence of compensatory mutations in the genetic backbone of patient-derived viruses at the moment of diagnosis that are able to restore viral RC.

## Discussion

In this study we strived to explain the *in vivo* persistence of the majority of TDRM in patients diagnosed with a drug-resistant HIV-1 variant. We selected patients diagnosed with HIV-1 containing limited profiles of TDRM, which are the most frequently transmitted variants as shown by large epidemiological studies [2,4]. In our patients, the vast majority of TDRM persisted for at least a year and up to eight years, confirming observations from previous studies that except for M184V/I, TDRM generally persist for longer than one year [10,13-25].

To explore the potential role of viral RC in persistence of TDRM, we investigated the impact of TDRM on the RC. *In vitro* determination of RC in PBMCs demonstrated that most site-directed mutant viruses harboring 1–2 frequently observed TDRMs had a reduced RC. However, in line with *in vivo* persistence the majority of patient-derived viruses had a higher RC than the corresponding SDM viruses. This suggests that polymorphisms, which may be present at baseline, can act as compensatory mutations. Our extensive sequence analysis demonstrated limited evolution on polymorphic positions, suggesting that in many transmitted HIV variants harboring TDRM compensatory mutations are already present at diagnosis.

Of the investigated site-directed mutant viruses, T215Y is known to evolve to atypical or partial revertant amino acids. Such alternative amino acids are known to confer limited impact on viral RC [9,18,31], which is in line with the observed persistence of revertant and atypical T215 variants in our and other studies [10,13,15-25].

Interestingly, when present as a SDM in the commonly used lab strain HXB2, K103N decreased the RC in our experiments although this NNRTI-related mutation has been described to have a low impact in several [32-34] but not all [35] previous studies. This discrepancy may be due to the use of different assays or differences in replication caused by polymorphisms in lab strains. Indeed, the RC of patient-derived K103N was similar to WT virus, indicating that polymorphisms can restore viral RC. This may explain the *in vivo* persistence of K103N in our and previous studies [10,21].

Several papers have described the impact of some drug resistance mutations on the RC of HIV-1 [16,32,33,35]. To our knowledge, the viral RC of frequently observed protease and RT TDRM has never been compared. Our data reveal that site-directed mutations at position 46 in protease have the most severe impact on RC.

Lack of reversion of the TDRM could be explained by a relatively small viral population size resulting in limited evolution. However, the median plasma HIV-RNA level of the included patients is similar to the HIV-RNA generally observed for newly diagnosed patients in the SPREAD programme [3]. Furthermore, although viral evolution was limited, in all transmitted viral variants changes at polymorphic sites were observed, indicating that replication could result in molecular evolution.

Certain resistance mutations such as M46I in protease have been described to decrease recognition of epitopes by certain HLA types [36]. As a result, also the immune system may affect viral evolution and persistence of TDRM. However, the majority of frequently observed TDRM may not impact or can even enhance recognition of epitopes [36,37] and as such, it is unlikely that the immune system is the major driving force behind persistence of all TDRM.

We previously hypothesized based on an extensive literature study that the lack of reversion is related to the RC of transmitted HIV-1 variants harboring TDRM [9]. The currently described data confirms that TDRM may persist due to a high RC of the transmitted HIV-1 variant. Alternatively, the selection of additional mutations may restore the RC or result in compensatory fixation [30,38]. This important role of polymorphisms was supported by the differential impact of TDRM in the presence of patient-derived genetic background compared to site-directed mutants. For all but one investigated frequently observed TDRM, *in vitro* RC of patient-derived virus was higher than the corresponding SDM. A striking example is M46L. Although the single presence of M46L in HXB2 causes a large decrease in viral RC, this defect in RC is largely restored when M46L is present in a patient-derived genetic background.

M41L is one of the most frequently observed TDRM, and is an intriguing example emphasizing the impact of the genetic background on RC. As a single mutation, M41L in the background of wild type virus HXB2 decreased the RC. This decrease was even more pronounced in the genetic background of pM41L, which was specifically selected for this study because of the absence of known compensatory mutations V60I and S162A [30]. In sharp contrast, pM41L + T69S + L210E + T215S, the patient-derived virus with an extensive profile containing a M41L in the presence of the compensatory mutation V60I had a similar RC as wild type virus [30].

In addition, compensatory mutations may be observed outside the target gene of the antiviral compound. It has been demonstrated that mutations in *gag* may help to compensate the reduced protease activity conferred by resistance mutations in the protease itself [39]. Unfortunately sequencing of *gag* is usually not included in routine genotyping within Europe, impeding investigation of a potentially compensatory role of *gag* in this study. For RT, compensatory mutations may also be present in the connection domain [40], which again is not included in routine genoptyping.

For only a subset of patients we had laboratory evidence of recent infection. We cannot exclude that patients were initially infected with a viral variant harboring a more extensive resistance profile and that some of these mutations had reverted before the patients were diagnosed. As such, the observed limited evolution of *pol* may be a result of viral adaptation before diagnosis or may even have taken place in previous hosts. By using a more sensitive sequence method, we might have been able to increase the detection of TDRM in the included patients [11]. However, we have previously used ultra-deep sequencing to investigate the quasispecies in plasma of patients who were newly diagnosed with an HIV-1 variant harboring a single NRTI-related resistance mutation. In most patients we were unable to detect viral minority variants harboring more extensive resistance profiles in the plasma, which may be suggestive of infection with a circulating HIV-1 variant harboring a limited resistance profile [41]. It is not unlikely that onward transmission of highly stable HIV-1 variants harboring limited resistance profiles greatly contributes to the current epidemic of transmitted drug resistant HIV-1 variants. Indeed, phylogenetic studies have demonstrated that onward transmission by untreated patients is a major source of transmission of drug-resistant HIV-1 [42-44].

It is of great clinical importance to be able to distinguish whether transmitted drug resistant HIV-1 variants harbor complex but partially reverted resistance profiles or circulating HIV-1 variants containing limited resistance profiles. For the frequently observed NNRTI-resistance mutation K103N, it is well-known that it causes high levels of resistance against all first generation NNRTIs [45,46]. Even when K103N is present as minority variant, it can contribute to therapy failure [11]. Fortunately, the recently approved second-generation NNRTIs remain active against HIV-1 harboring a single K103N [47,48]. In contrast, we have demonstrated that the NRTI-related M41L in RT has limited impact on selection of resistance against currently used NRTIs [49]. M46I/L or L90M as a single TDRM in protease may cause low level resistance to commonly used protease inhibitors such as lopinavir.

## Conclusion

In conclusion, we confirmed persistence of the most frequently observed TDRM. All transmitted HIV-1 variants harbored additional polymorphisms, with limited selection of additional mutations. Limited reversion of TDRM is in concordance with the high *in vitro* RC of patient-derived viruses harboring TDRM. As SDM viruses with the same TDRM as patient-derived viruses have a lower RC *in vitro*, we propose that polymorphisms that function as compensatory mutations (partially) restoring viral RC explain the *in vivo* persistence of TDRM. The stability of transmitted drug resistant HIV-1 variants can facilitate onward transmission of these viruses.

## Methods

### *In vivo* evolution

#### Ethics statement

Ethical requirements differ between countries according to national legislation. In countries where a national surveillance system was established, legally no informed consent was needed. In other countries, approval was obtained by the institutional medical ethical review committees. All data were anonymized at national level.

#### Patients

Patients from four countries participating in the SPREAD-programme (Belgium, Greece, the Netherlands, Slovenia) were included. For all included patients, a baseline genotypic resistance test performed on a plasma sample within three months after diagnosis of HIV-1 infection revealed at least one mutation on a position associated with transmitted drug resistance as described in the mutation list as recommended by the WHO [26]. Patients were included on the basis of sample availability; a base line sample and a sample one year (10–14 months) later. If available, a sample at later time points were included. All included patients were at least 18 years of age and not exposed to antiretroviral therapy during the study period.

#### Sequence analysis

Genotypic resistance tests were performed by population sequencing of the viral protease and part of reverse transcriptase using commercially available assays or in-house methods covering at least amino acids 4–99 of protease and amino acids 30–249 of RT. All laboratories collaborated in the quality control program of ESAR to ensure high quality genotypic data [3,4]. HIV-1 subtype was determined using REGA 2.0 [50]. To investigate evolution, the p-distance and the ratio of the proportions of synonymous and nonsynonymous substitutions (dS/dN ratio) were calculated using MEGA 5.05. The p-distance is the proportion of nucleotides between two sequences that has been changed. The dS/dN ratio, a measure of selection pressure [51], was calculated with the Nei-Gojobori method and statistically tested with a Z-test.

### *In vitro* determination of replicative capacity

#### Virus panel

Mutations M46I, M46L and L90M in protease and M41L, M41L + T215Y and K103N in RT were introduced in HXB2 by site-directed mutagenesis using the previously described vector systems CP-MUT and NRT-MUT [52] and the following primers: M46I 5′-GGA AAC CAA AAA TAA TAG GG-3′ (HXB2 nucleotides 2380–2396), M46L 5′-GGA AAC CAA AAC TGA TAG GG-3′ (HXB2 nucleotides 2380–2396), L90M 5′-GAA ATC TGA TGA CTC AGA TTG-3′ (HXB2 nucleotides 2511–2532), M41L 5′-ATT TGT ACA GAG CTG GAA AAG GAA G-3′ (HXB2 nucleotides 2658–2682), K103N 5′-GTT ACT GAT TTG TTC TTT TTT AAC CC-3′ (HXB2 nucleotides 2844–2869), T215Y 5′-TGTCTG GTG TGTAAA GTCCCCACC-3′ (HXB2 nucleotides 3181–3204).

Baseline patient-derived viral protease genes harboring M46I, M46L, L90M or I54V + V82A + L90M or the N-terminus of RT containing M41L, M41L + T69S + L210E + T215S or K103N were introduced into HXB2 using the same vector system [52].

Clones were obtained and sequence analysis was performed to verify resemblance to population sequences. Subsequently, at least three recombinant virus stocks were generated by Lipofectamine 2000 (Invitrogen) transfection of HEK293T cells according to manufacturer’s guidelines. TCID_50_ was determined by end-point dilution in MT2 cells, demonstrating similar replication in this T cell line in all cases. A random clone was selected and quantified by p24 ELISA (Aalto Bioreagent, Dublin, Ireland) for the RC analysis.

#### RC analysis

PBMCs were isolated from HIV-seronegative blood donors by Ficoll-Paque density gradient centrifugation and stored in liquid nitrogen until use. To minimize differences between batches caused by variation between donors, each batch of PBMCs consisted of five combined donors. The RC of the virus panel was determined by infecting 5×10^6^ phytohaemagglutinin-stimulated (2 mg/L) donor PBMCs with the equivalent of 40 ng HIV-1 p24 for two hours. Subsequently, cells were washed twice and maintained for 14 days in RPMI1640 with L-glutamine (BioWhittaker), 10% fetal bovine serum (Biochrom AG), 10 mg/L gentamicin (Gibco) and 5 U/ml IL-2. Cell-free supernatant was harvested daily for monitoring of the p24 production. The RC of either the SDM-viruses or the patient-derived viruses was compared to the RC of control viruses (WT, HIV-M184V, −M184I and –M184T). By comparing viruses containing only the mutation(s) or gene of interest in the exact same HIV-WT background, it is possible to determine the impact of these relevant mutation(s) or genes on viral RC. For all viruses, replication curves were performed in four biological replicates divided over two independent experiments. The mean p24 production of two replicates within representative experiments are indicated in Figure 1A-C for protease and 1D-F for RT. Figure 1G represents the median p24 production relative to HIV-WT of all four replicates on day 7 post infection.
